# Why peer assessment helps to improve clinical performance in undergraduate physical therapy education: a mixed methods design

**DOI:** 10.1186/1472-6920-14-117

**Published:** 2014-06-13

**Authors:** Marjo JM Maas, Dominique MA Sluijsmans, Philip J van der Wees, Yvonne F Heerkens, Maria WG Nijhuis-van der Sanden, Cees PM van der Vleuten

**Affiliations:** 1 HAN University of Applied Sciences, Department Allied Health Studies, Kapittelweg 33, 5425 EN Nijmegen, The Netherlands; 2 Radboud University Medical Center, Scientific Institute for Quality of Healthcare, Geert Grooteplein 21, 6525 EZ Nijmegen, The Netherlands; 3 Zuyd Hogeschool, Department Educational Research, Heerlen, The Netherlands; 4 Dutch Institute of Allied Health Care, Amersfoort, The Netherlands; 5 Maastricht University, Department of Educational Development and Research, Faculty of Health, Medicine and Life Sciences, Maastricht, The Netherlands

**Keywords:** Peer assessment, Peer feedback, Self-assessment, Clinical performance

## Abstract

**Background:**

Peer Assessment (PA) in health professions education encourages students to develop a critical attitude towards their own and their peers’ performance. We designed a PA task to assess students’ clinical skills (including reasoning, communication, physical examination and treatment skills) in a role-play that simulated physical therapy (PT) practice. Students alternately performed in the role of PT, assessor, and patient. Oral face-to-face feedback was provided as well as written feedback and scores.

This study aims to explore the impact of PA on the improvement of clinical performance of undergraduate PT students.

**Methods:**

The PA task was analyzed and decomposed into task elements. A qualitative approach was used to explore students’ perceptions of the task and the task elements. Semi-structured interviews with second year students were conducted to explore the perceived impact of these task elements on performance improvement. Students were asked to select the elements perceived valuable, to rank them from highest to lowest learning value, and to motivate their choices. Interviews were transcribed verbatim and analyzed, using a phenomenographical approach and following template analysis guidelines. A quantitative approach was used to describe the ranking results.

**Results:**

Quantitative analyses showed that the perceived impact on learning varied widely. Performing the clinical task in the PT role, was assigned to the first place (1), followed by receiving expert feedback (2), and observing peer performance (3). Receiving peer feedback was not perceived the most powerful task element.

Qualitative analyses resulted in three emerging themes: pre-performance, true-performance, and post-performance triggers for improvement. Each theme contained three categories: learning activities, outcomes, and conditions for learning.

Intended learning activities were reported, such as transferring prior learning to a new application context and unintended learning activities, such as modelling a peer’s performance. Outcomes related to increased self-confidence, insight in performance standards and awareness of improvement areas. Conditions for learning referred to the quality of peer feedback.

**Conclusions:**

PA may be a powerful tool to improve clinical performance, although peer feedback is not perceived the most powerful element. Peer assessors in undergraduate PT education use idiosyncratic strategies to assess their peers’ performance.

## Background

Modern education in health professions aims at the development of reflective practitioners, capable of self-directing their professional development before and after graduation. Health care practitioners need to keep up with demands for improved quality of care and patient outcomes [1]. Peer Review is one of the strategies that health care practitioners apply for professional development, for upholding professional standards and to be accountable to stakeholders in health care [2]. Peer Assessment (PA) is a structured variant of Peer Review that can be described as the process whereby participants of similar status evaluate the performance of their peers and give quantitative and/or qualitative feedback. The strategy targets the development of a mutual accepted quality standard of performance by introducing peers with the ‘assessor’ or ‘auditor’ perspective. The PA approach implies that professional development is a shared responsibility and that individuals, teams and organizations may profit from the learning outcomes [3]. PA has become increasingly popular in Health Professions educational programs to encourage students to develop a critical attitude towards their own and their peers performance anticipating on lifelong quality improvement demands in clinical practice. A study of Sluijsmans [4] showed that students in higher education, who are trained to critically reflect on the performances of their peers, simultaneously develop self-assessment skills that might help them to self-direct their learning process. Research has shown that health care professionals have a limited ability to accurately self-assess their level of professional competence [5]. Self-assessment alone appears not to be a reliable source of information to identify shortcomings in clinical performance because practitioners tend to systematically over- or underestimate their level of competency [6,7]. The development of adequate self-perceptions requires additional information from external sources and comparing information with a performance standard [8]. Peers who are adequately trained in their peer assessor role, might provide the missing information to inform self-assessment and might uncover improvement areas that would remain undiscovered by self-assessment alone [9].

PA in health professions education is applied with different educational goals and implemented in various educational formats [10-12]. Gielen [13] distinguishes two main goals of PA: PA as an ‘assessment tool’ and PA as a ‘learning tool’. PA as an assessment tool refers to the ability of students to reliably and validly assess their peers. Most research on PA has conceived PA as an assessment tool. Peer judgment is either compared to faculty judgment or self-reports and the quality of PA is determined by a criterion validity approach [12,14-17]. This concept of PA is not applicable when PA is intended to inform self-assessment and improve performance. When PA is viewed as a ‘learning tool’ it aims to provide students with relevant improvement feedback [18]. In contrast with staff assessment, peer feedback is built up from multiple sources of information [19]. The quality criterion for PA as a learning tool can best be described by the concept of ‘consequential validity’, referring to the impact on student learning outcomes [13,20-22]. The majority of studies on the impact of PA on learning in health professions education report positive effects [12]. These studies however mainly focus on professional behavior such as rule-based adherence to behavioral norms, rather than (hands-on) clinical examination and treatment skills [15,23-26]. When it comes down to PA of clinical performance, validity evidence is scarce and limited to the medical domain [12,16,27-29]. However, diagnosis and treatment belong to the core business of health care practitioners and performance gaps might affect patient safety and intervention outcomes in the end [1]. The implementation of PA of clinical performance in undergraduate health professions education is therefore desired. Research showed that one of the determinants of effective PA processes is training in PA skills [10,11]. When students are trained to adequately assess their peers and to provide meaningful improvement feedback, they might be well prepared to ‘audit’ their colleagues after graduation. Yet, we do not know how PA impacts on the improvement of clinical performance and validity evidence is needed.

We designed a complex PA task that aims to facilitate students to improve their clinical performance prior to work placement. Clinical performance included reasoning skills, communication skills and practical physical examination - and treatment skills. A mixed methods approach was taken to analyze the following research questions.

How does the PA task impact on the improvement of clinical performance in the perception of PT students?

1. *Which elements of the PA task have a powerful impact on learning and what are factors conditional for learning?*

2. *Why do students perceive these task elements as powerful?*

## Methods

### Study design

A qualitative approach was used to explore students’ perceptions of the PA task and the distinct PA task elements. A quantitative approach was used to identify the elements that have the strongest impact to strengthen the qualitative data.

### Context and participants

This study was conducted within the Department of Physical Therapy at the HAN university of Applied Sciences in the Netherlands in 2008. The PA task was part of the course ‘Physical therapy in primary care-2’ that was offered in the second year of the bachelor program, prior to work placement. The course consisted of two blocks of seven weeks. Participation in the PA task was compulsory, but the use of the PA results was formative. Ten groups of twelve students completed the task (n = 120). A purposive sample of 12 students was invited for interviews by MM using e-mail. Sampling was based on maximal variation in groups, gender and nationality.

### The design of the PA task

The PA task was designed as an authentic, complex learning task. Performance of clinical skills was observed and evaluated by peers in a role play that simulated physical therapy practice. The task was pre-tested after the first block of seven weeks and evaluated in a pilot study including student interviews.

In the PA sessions, students alternately performed in three roles: physical therapist (PT) role, assessor role, and patient role. At the beginning of the role play, each group member received a short written clinical case. The simulated patient received an additional role description. In the PT role, students demonstrated relevant examination or intervention skills. In the assessor role, students provided immediate face-to-face oral feedback, written feedback and scores. In the patient role, students simulated the written clinical cases according to the role description and provided feedback afterwards. Table 1 shows the task procedure. Expert assessors (teachers) took part in the PA session in the role of end assessor, providing additional feedback if necessary and only when all peer feedback had been collected. Students were provided with a manual that allowed them to prepare the task in advance. It contained the learning goals of PA, a structured task procedure and a set of short clinical cases, according to the *key-feature* concept [30]. These cases served as PA material to enhance the transfer of knowledge and skills to new problems [31]; students could choose to study them in advance or not. The manual also provided an assessment form, consisting of four global performance indicators that could be scored on a 7-point Likert scale and an open field for written comments. The form was validated in a previous study [32] and adapted to PA. Students were instructed in giving high-quality improvement feedback during the pilot and feedback guidelines were included in the manual.

**Table 1:** Peer assessment task procedure

**Time**	**Task**	**Therapist role**	**Patient role**	**Assessor role**
5 min.	Study written clinical case and clinical assignment	x	x	x
	Study simulation role information		x	
3-5 min.	Explain choice for intended examination or treatment	x		
8-10 min.	Perform examination – or treatment task	x		
3-5 min.	Fill out assessment form			x
4-5 min.	Provide oral improvement feedback		x	x
	Comment on feedback	x		
25-30 min.				

Each peer group consisted of 6–7 students, which has showed to be an effective size for this purpose [15,33]. The task was presented prior to the final summative assessment of clinical performance that was decisive for the entrance of work placement. When the task was completed, students wrote a reflection report, using the PA feedback and scores. The reflection report served as participation evidence in their portfolio.

### Data collection

The PA task was analyzed and decomposed by the method of Janssen-Noordman [34] to identify constituent task elements that might trigger improvement. The analysis of the PA task in task elements was discussed by a team of five experts until consensus was reached, and was validated by 12 participating students in the pilot study. Task analysis resulted in 13 task elements (Table 2).

**Table 2:** Ranking of task elements according to perceived impact on performance improvement

**Task**^**a**^	**Constituent**^**a**^**task**	**Student (S)**														**N**^**b**^	**Sum**	**R**^**c**^
		**S1**	**S2**	**S3**	**S4**	**S5**	**S6**	**S7**	**S8**	**S9**	**S10**	**S11**	**S12**	**S13**	**S14**			
Prepare Task	**Study manual**		9											4		2	13	**9**
	**Study cases**	8	8	9	4		9	8	4		4	6		7		10	67	**5**
Perform in PT role	**Give performance**	9	6	8	9	9	8	9	8	7	9	8	9	8		13	107	**1**
	**Receive peer FB**	5		6	6	7	3	3	5	5	7	7	7		8	12	69	**4**
	**Receive expert FB**	6		7	7	8	2	4	6	6	8	9	8	5	9	13	85	**2**
	**Receive patient FB**									4						1	4	**12**
	**Receive score**							2		3					4	3	9	**10**
Perform in assessor role	**Observe performance**	4	4	4	5	6	7	7	9	9	5	4	5		6	13	75	**3**
	**Give oral FB**		7	3		5	6	6	7	8		3	4		5	10	54	**6**
	**Give written FB**					4										1	4	**12**
	**Give Score**		3				5									2	8	**11**
**Perform in patient role**		7	5		8		4	5		2	3	5		6		9	45	**7**
**Write reflection report**				5		3	1	1		1	6		6	9	7	9	39	**8**

^a^Tasks presented for ranking are bold face.

^b^N of students that selected the task for ranking.

^c^Final rank order.

A semi-structured interview guide was designed on the basis of pilot study results. Interviews were conducted by the principal researcher (MM) while notes were taken by a research assistant (EL). Students were invited for interviews by purposive sampling, aiming at maximal variation in groups, gender and nationality. The distinct task elements of the PA-task were presented on separate cards at the beginning of the interview. Students were asked to select the elements perceived to have a powerful impact on performance improvement and to rank the selected elements from highest learning value (rank 1) to the lowest. Task elements that were not selected were left out of the ranking procedure. Subsequently, students motivated their choices. Interviews were audio-taped after informed consent was obtained of each participant, and were transcribed verbatim. Interviews lasted between 45 and 60 minutes. Data collection was ended when saturation was reached, meaning that additional sampling would not contribute to new findings.

### Data analysis

Ranking results of each selected task element were entered in IBM SPSS Statistics 20.0. Ranking numbers were re-coded into scores, awarding the first rank with the highest score and, the last rank with the lowest score. Frequencies were described and sum scores were calculated for each task element.

Five transcripts were studied and relevant quotes were coded independently by MM and EL. PA task elements were used as a-priori categories (defined in advance) to structure the data in a way that research question 2 was directly addressed. We followed the method of Nigel Kings’ template analyses that showed to be an adequate method for this purpose [35]. Codes were discussed until consensus was reached and a coding scheme was created. Subsequently all transcripts were analyzed by MM and EL. New themes emerged from the data by constant comparison of codes and categories. A data matrix was constructed that crossed task elements (a-priori categories) with themes and categories that emerged from the data [36]. Finally, a conceptual model of how PA affects learning was designed that fully fitted the data. To enhance credibility, the analysis process was checked by a project consultant (JB) that was specialized in qualitative research methods. Disagreements were solved by discussion until consensus was reached. A member checking procedure was carried out among all interviewed students.

### Ethical aspects

This project received approval from the Faculty board of Han University of Applied Sciences. All participants volunteered to participate and anonymity and confidentiality was assured. They signed an approved consent form.

### Statement

This study adhered to the RATS guidelines for qualitative research: http://www.biomedcentral.com/authors/rats.

## Results

Quantitative analyses showed that all 13 presented task elements were selected, assigned to 12 ranks (2 tasks on rank 12). Table 2 shows that the perceived learning value of distinct task elements varied widely among students. The majority of students perceived ‘performance in the physical therapist role’ as the most valuable task element (1) followed by receiving expert feedback (2), and observing peer performance (3). Receiving peer feedback was not perceived the most powerful task element.

Twelve interviews were conducted representing all groups and two additional interviews were needed to reach data saturation. Qualitative analyses resulted in three major themes that explained how the PA task impacts the improvement of clinical performance: 1) pre-performance triggers, referring to the anticipatory cognitive motivators related to students' perceptions of the learning environment that were conceived as *feed forward*, 2) true-performance triggers, referring to the vast array of inputs elicited by performing the task that can be conceived as *internal feedback*, and 3) post-performance triggers, referring to knowledge of performance and knowledge of results that was conceived as *external feedback*. Each theme contained three categories: a) learning activities, b) learning outcomes, and c) conditions for learning. The results are summarized in Table 3.

**Table 3:** Summary of learning activities, learning results and conditions for learning related to distinct task elements

**Triggers**	**Task elements**	**Learning activities**	**Learning results**	**Learning conditions**
**Pre-performance triggers**	Study manual	Self-study	Knowledge of performance standards	
*Feedforward*	Study cases	Practice	Reduction of performance anxiety	
**True-performance Triggers**	Perform PT role	Cope with anxiety triggers	Increased self-confidence	
		Apply learning in new context	Awareness of improvement areas	
*Internal feedback*				
		Reason aloud		
		Act methodically		
	Perform in patient role	Empathise with patient problem		
	Observe performance	Matching intended performance with observed performance	Re-design of intended performance	
		Modelling	Increased self-confidence	
			Knowledge of alternative performance	
			Awareness of improvement areas	
	Give oral feedback	Study criteria	Insight in performance standards	
	Give written feedback	Structure information		
	Give score	Empathize with peer		
		Explicit views		
**Post- performance triggers**	Receive peer feedback	Ask for clarification	Knowledge of performance from different perspectives.	Peer is well prepared and has sufficient case-specific knowledge
*External feedback*		Analyse information		
			Knowledge of alternative performance	Feedback is critical, specific, concrete, reveals strength and weakness and contains improvement suggestions
			Awareness of improvement areas	Feedback meets learning needs
				Peer is involved in learning process
	Receive expert feedback		Knowledge of expert standards	Expert allows for discussion over criteria
			Validation of peer feedback	
	Receive patient feedback		Knowledge of patient perceived aspects	Sufficient case-specific knowledge role-player
	Receive score	Compare sum scores and domain scores	Knowledge of results compared to the group	Peer has enough courage to give low scores when necessary
**Reflection**	Write reflection report	Select feedback		
		Relate information to prior feedback		
		Create new learning goals		

### Pre-performance triggers

#### Expectations and personal goals

Students had different expectations of PA that colored their views. The majority of students viewed the assessment as an appropriate training prior to their summative assessment or their future professional practice. However, some students had little confidence in the assessor qualities of their peers.

“My expectations were not that high, because the group in which I worked was not so good. Yes, my expectations of the first PA were confirmed and so were my expectations of the second assessment. I needed to show that I participated to meet portfolio demands, and I did, but I was not satisfied … the feedback was superficial and I was glad that there was an expert”.

#### Study manual and clinical cases

Students felt triggered to study cases prior to assessment. The cases were new and represented a sample of the context in which prior learning needed to be applied. Students were motivated either out of fear of encountering unexpected demands or simply out of curiosity.

“In the previous PA I was not prepared and I stood there a bit nervous, waiting for what would happen. Now I have prepared the cases, I know what I can do and that feels much better”.

### True-performance triggers

#### Perform in the physical therapist role

The transfer of learning into a new context was perceived as challenging. Students embraced the opportunities for new clinical encounters. They were triggered by both curiosity and eagerness.

Firstly, the PA context differed from the learning context because their actions were being watched. They needed to cope with anxiety triggers common in this type of performance.

“…You have an assignment and 12 eyes are watching you. You must also be able to perform under that pressure. You do not want to blunder before your classmates”.

Successful coping resulted in increased self-confidence.

“…When you’re insecure, but you have to perform the task, and then it turns out that you performed well, then you feel strengthened. Like I’m not someone that knows nothing and that feels good”.

Secondly, the presence of a (simulated) patient required transfer of knowledge and skills to the specific content of the patient problem and the specific patient needs. Organized domain specific knowledge needed to be combined with new, unexpected information.

“..yes, in class you practice without a patient. When your skills are good, you do not bother. In PA you have to deal with a patient”.

Students were confronted with having little professional language available to explain to their peers what they were planning to do and why. They were triggered to reason aloud.

“….. usually it is in your head, but now you have to argue aloud. Normally you don't explain why you choose for a certain clinical test, but when you're asked, yes, you need to answer”.

Similar to clinical reasoning aloud, the majority of students felt triggered to transform declarative knowledge (knows) into procedural knowledge (knows how) and performance (shows how).

“…and you do it sequentially, not just in pieces as in class, but the whole thing, in which all those pieces have to be glued together…”

Although students perceived performance in the physical therapist role as the most valuable task element, data analyses showed that this learning experience cannot be separated from learning in other roles.

“Critical appraisal of a peer’s performance, is easier said than done. When you perform in the assessor role, you actually act as a physical therapist”.

Students either reflected or anticipated on their performance in the physical therapist role by continuous comparison of personal performance with peer performance and personal views with peer views as shown in the following paragraphs.

#### Perform in the assessor role

In observing their peers, students reported learning activities taking place on a more or less unconscious level as well as to learning activities which can be clearly described. The unconscious level refers to mirroring and matching the observed performance to the virtual image of one's own performance. The more conscious level refers to using the peer as a ‘model’ to improve their own clinical performance. Although student reports come across both levels, some efforts are made to make a distinction in learning activities:

#### Matching

“..you are very focused on looking at what someone is doing. You learn a lot by just watching. Actually, when you observe someone else, you imagine how you would act yourself. Like, how could I stand there, how would I do that?”

#### Modelling

“For example....you see certain actions, you make notes, and you might try them out later”.

When giving peer feedback, their personal picture was compared to peer views. Students were challenged to structure, summarize and communicate their (implicit) observations. Giving feedback prompted discussion, providing them a deeper understanding of performance criteria.

“I express how I see it, how I think it should be done and there again you get a reaction. Also a kind of clinical reasoning actually”.

The peer assessor view appeared to develop during the assessment process and students became aware of their views by reasoning aloud.

“When I see what others are doing wrong, then I ask myself: ‘how am I doing that? And what is good?’ Then I'm going to ask the rest of the group: ‘how do you do that? And do you agree with the way this person did it?’”

### Post-performance triggers

#### Receive expert feedback

Being in the middle of the course, with the end-course assessment ahead, students wished to know what improvements they should make to meet the expert standards. Expert feedback contributed to the credibility of peer feedback that advanced acceptance of a peers’ judgment or advice.

“…hard to say …. a piece of approval so to speak. I need to be sure what I have to improve. Expert feedback is a kind of confirmation of peer feedback”.

#### Receive peer – and simulated patient feedback

Most students valued peer feedback because of its variety and completeness. Students who were reluctant in asking for feedback during the course, mentioned the advantage of this task to obtain exclusive feedback.

“Yeah, I'm pretty insecure. I like it when someone specifically looks at me, that I receive personal attention. I easily push myself on the side”.

The involvement of students in the learning process of their peers was generally considered an advantage. Peers were able to keep a record of errors what expert assessors usually do not and that enhanced peer feedback credibility and acceptance.

“....yes, maybe it is like you usually practice together and they know you better; they know when you make mistakes by nervousness, they know your positive and negative sides. And if you're using the same group again in PA, they know what you had to learn”.

Yet this was not an argument in favor of feedback being just nice; students agreed on conditions for learning from peer feedback. They reported that the acceptability and the usefulness of peer feedback heavily relied on appropriate task-specific knowledge, sufficient task preparation and enough peer assessor skills. In addition, feedback should be critical, revealing strengths and weaknesses and should contain improvement suggestions. Even judgmental feedback was mentioned. So-called ‘soft feedback’ consisting of global comments on communicational aspects, missing any connection to clinical performance, was widely rejected.

“Critical feedback. May also be judging. Empathy is important, but I do not like someone to just repeat what has been said with a very sweet voice”.

Students however did not ask for the feedback they wanted in advance. Instead they complained afterwards of receiving feedback that did not meet their expectations.

“How I communicate with patients, that I know by now. Clinical reasoning, that is currently important to me. I want to know for myself why I do the things I do and I want to be able to explain that when anyone else asks me to”.

Give and receive written feedback and scores.

Elaborative oral feedback was preferred over written feedback and scores. Received scores were not perceived reliable, because peers lacked objectivity from an interpersonal perspective. For the same reason some students felt reluctant in giving scores. They, however, reflected on scores in a meaningful way by trying to find a certain convergence in the domains that needed improvement.

“Suppose I have e.g. 20 points and someone else has 30, so I'd always look for a category where the difference is. The final score does not tell me so much. Although it is good to have a score for each category to see where you still need to work on something”.

#### Write reflection report

Writing reflection reports is perceived with mixed feelings. For some students it was helpful to self-direct their learning process, others perceived the (compulsory) task as unnecessary work load, especially for immigrants.

“….so I think .. well I finished my assessment, I have received my feedback and now I also have to write it down. Actually, I do know enough. Why do that once again?”

## Discussion

**Figure 1:**
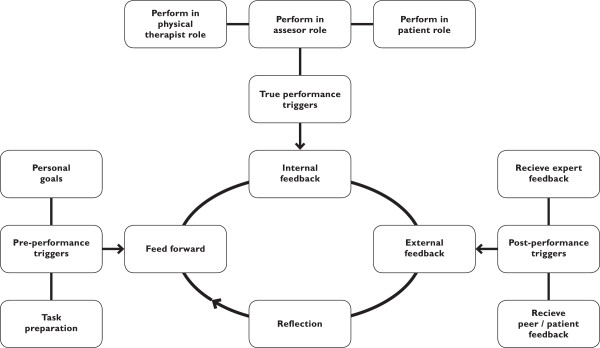
Conceptual model of information processing in peer assessment of clinical performance.

Our results show that the PA task contains a variety of elements that have a positive impact on the improvement of clinical performance. We developed a conceptual model, based on our results, that fully fits the data and that reflects how information is processed in PA to inform self-assessment (Figure 1). The model shows that learning begins by anticipating the PA context as well as the PA content (pre-performance triggers), described as the ‘backwash effect’ of assessment [37] or the ‘feed forward function’ of assessment [38].

Following the model, performance in different roles is the next phase. Performance in the physical therapist role was perceived to have the strongest impact on learning. This finding is in contrast with the general assumption that ‘peer feedback’ determines the impact of PA on learning [39]. However, social learning theory might provide an explanation. It emphasizes the importance of mastery experiences for performance improvement. Students needed to cope with anxiety triggers related to the context and content of PA. Successful coping resulted in increased self-confidence and awareness of strength and weaknesses. Studies of Bandura [40,41] show that mastery experiences are the strongest source of information for the development of self-efficacy beliefs and self-efficacy beliefs contribute significantly to the level of motivation for performance improvement. Rush [42], who studied the impact of PA on the performance of clinical skills in undergraduate nursing education, also reported high perceived learning value of performance in the nursing role, and found increased *self-confidence* as a dominant finding. Apparently, personally perceived mastery evidence is more powerful than mastery evidence provided by peers in undergraduate education. The PA task challenged, or even forced, students to transfer knowledge and skills to a new application context which they apparently did not do spontaneously and that might be the key-feature of PA for improvement of clinical performance. Simons [43] argues that learners oftentimes do not and cannot know ‘what’ knowledge and skills need to be transferred and to ‘what’ new context, so they need help. Successful transfer of learning depends on the distance between the learning context and application context. A short distance (near transfer) refers to solving *new* problems in the *same* context. A long distance (far transfer) refers to solving *new* problems in a *new* context. Apparently, the PA task construed a transfer gap that was ‘near’ enough to successfully bridge, but ‘far’ enough to be challenging. In effect, the task was in the ‘zone of proximal development’ as described by Vygotskiĭ [44]. Apart from these considerations that aimed to explain the superior perceived learning value of performance in the PT role, it should be noted that data analysis showed an interaction effect between performance in different roles. Learning experiences in the PT role may have been strengthened by performance in other roles and that may have influenced students’ choice for the most valuable task element.

Concerning performance in the assessor role, we found results that were not reported by prior research. Peer assessors apply strategies to assess their peers that differ considerably from experts. Firstly, from a stakeholder perspective, students have different interests in observing the performance of their peers than expert assessors. Students have a need to improve their own performance whereby experts presumably do not. Thus peers may focus on different aspects than experts. Secondly, students obviously do not focus beforehand on critical features expressed in pre-determined criteria like expert assessors do. They use their peers, although not consciously. They ‘match’ and ‘model’ the observed performance to the image of their own performance. Research has revealed that the human motor system has mirroring capacity and is activated by observing motor actions made by others [45]. By mirroring the observed action, the brain is prepared to execute the same action. Calvo-Merino et al. [46] studied the differences in mirroring activity between watching an action that one has learned to do and an action that one has not. They compared experts in classical ballet with experts in capoeira (a traditional dance) observing both dancing styles and showed that mirroring activity is more powerful when expert dancers viewed movements that they had been trained to perform compared to movements they had not. The foregoing might explain students’ engagement with observing their peers’ performance. Although it is unknown how expert assessors actually view the performance of their students, it may be assumed that the ‘virtual image’ of the expert is different, as it is built and shaped by experience [47]. Thirdly, students observe more than experts. They are involved with the learning process of their peers and have more detailed knowledge of their learning needs than expert assessors have.

Receiving feedback represents the next phase in the model. Students preferred expert feedback over peer feedback because experts represent the performance standard for summative decisions, which is obvious, because students depend on their judgment. Peer feedback, however, was valued because of its variety and its completeness and the involvement of peers in each other’s learning was perceived a positive condition for identifying improvement areas. This finding is supported by several studies on PA in the health professions domain [12,23-26,42], although some studies report reluctance of peers in giving face-to-face feedback [33,48].

Reflection represents the final phase in the model, referring to explicit conscious reflection on the PA-task that resulted in insight in strength and weaknesses and new learning goals. However, the perceived value of writing reflection reports was limited, which is understandable. Data show that reflection also occurred, although less explicitly, as a response to pre- and true-performance triggers as conceptualized by Schӧn’s model of reflective practice [49].

What this study adds to prior research is that peer judgment cannot be compared nor replaced by expert assessor judgments. However, the peer assessor view that develops during the PA process and the expert assessor view that represents the ‘golden standard’ may both provide students with rich just-in-time improvement feedback, built on multiple perspectives and connecting to their learning needs. Research on PA revealed that effective PA processes depend on training and experience [4,11,50]. When peers continue to compare their personal views to peer views, they might gradually develop an internalized and mutually shared quality standard of performance that enhances professional development for now and after graduation. Future research should determine whether experienced peer assessors converge in their performance judgments.

### Limitations

The generalizability of the qualitative data is limited. There is evidence that attitudes towards PA are gender- and cultural dependent [51] and that learning from PA depends on the PA-context, PA- content, and peer feedback preferences [10,11,19,22,28,39,51]. In addition, the generalizability of the quantitative data is limited because of the small sample size. It should be noted however, that the quantitative data were intended to strengthen the qualitative data and not vice-a-versa.

## Conclusions

The PA task contains a variety of elements that have a positive impact on the improvement of clinical performance. It triggers intended learning through peer feedback and reflection as well as unintended learning through matching and modelling a peer’s performance. PA might be a powerful tool to help students in bridging the gap between the learning context and the application context. Peer feedback however is not perceived the most powerful task element in undergraduate physical therapy education and peer assessors use idiosyncratic strategies to assess their peers’ performance.

## Competing interests

The authors declare that they have no competing interests.

## Authors’ contributions

MM contributed to study conception and design, sampling, analysis and interpretation of data, and drafting of the manuscript. DS contributed to the study design, data analyses and drafting of the manuscript. PW, YH critically revised the manuscript. MN and CV contributed to data analysis and critically revised the manuscript. All authors read and approved the final manuscript. All authors agree to be accountable for all aspects of the work in ensuring that questions related to the accuracy or integrity of any part of the work are appropriately investigated and resolved.

## Authors’ information

Marjo Maas (MSc) is a teacher at the HAN University of Applied Sciences, Department Allied Health Studies, a researcher and PHD student at Radboud University Medical Center, Scientific Institute for Quality of Healthcare, The Netherlands.

Philip van der Wees (PhD) is a senior researcher at Radboud University Medical Center, Scientific Institute for Quality of Healthcare, The Netherlands.

Dominique Sluijsmans (PhD) is associate professor at Maastricht University, lector at Zuyd Hogeschool and staff member at ICO Interuniversity Center for Educational Research, The Netherlands.

Yvonne Heerkens (PhD) is associate professor at HAN University of Applied Sciences, Research Center for Rehabilitation Work and Sports and program manager at the Dutch Institute of Allied Health Care, The Netherlands.

Maria Nijhuis – van der Sanden (PhD) is professor at Radboud University Medical Center, Scientific Institute for Quality of Healthcare, The Netherlands.

Cees van der Vleuten (PhD) is professor at Maastricht University, Department of Educational Development and Research, Faculty of Health, Medicine and Life Sciences, The Netherlands.

## Pre-publication history

The pre-publication history for this paper can be accessed here:

http://www.biomedcentral.com/1472-6920/14/117/prepub
